# Book Review

**Published:** 1927

**Authors:** 


### Reviews of Books.

**The Study of Medicine.** By Alexander Miles, M.D., F.R.C.S., LL.D. Pp. vii., 92. Edinburgh: Oliver & Boyd. 1925. Price 3s.—This brochure is intended to help the student to realise at the beginning of the medical curriculum the unity of his course and the interdependence of its several parts. It shows why in addition to a general education the medical student must have a working knowledge of chemistry, physics and biology, it points out the bearing of anatomy, physiology, pathology and pharmacology upon the practice of medicine, and finally discusses how the "professional" subjects, medicine, surgery, midwifery and the allied specialities, are applied in the interests of the patient, or, in certain branches, in the interests of the community. It is no bad book for the young student; it is a better one still for his teacher, growing old and perhaps fossilised in his own narrow layer of knowledge.

**What's Best to Eat.** By S. H. Belfrage, M.D. Pp. xi., 199. London: William Heinemann Ltd. 1926. Price 7s. 6d. —Everyone nowadays is interested in his food. As the author says, "The well-to-do community make of their meals a social function, and by the elaborateness of the menu seek to establish their social position." But he puts a sting in the tail of the sentence when he adds, "at the risk of their health." We should all be grateful if we could be taught how to be socially successfully without risk to our health. The only trouble about a book like this is that it seems almost to postulate the desirability of being aware of the metabolic and mechanical processes of digestion. Whereas we believe that the happiest individuals don't even know how many stomachs or colons they possess. In this volume there is a considerable amount of fact about food values and the like, combined with a good deal of speculation about the dire effects of constipation. But we really must pause a moment before the sentence, "It is no exaggeration to say that constipation may well bring the downfall of civilisation in its train." We expect to have our flesh made to creep, but perhaps the story is reserved for a subsequent volume. We read on and discover that we might individually develop degenerative conditions of the liver, kidneys and skin, but the threatened disaster to civilisation is not revealed. The practical supplement by Lucy H. Yates, M.C.A., contains some attractive cookery recipes. It would be almost worth while risking obesity to live daily on her " brown scones."

**The Elements of Medical Treatment.** By ROBERT HUTCHISON, M.D., F.R.C.P. Pp. vii., 163. Bristol: John Wright & Sons Ltd. 1926. Price 7s. 6d.—When Dr. Hutchison writes a book it is sure to be both interesting and instructive, and this is no exception. For several years the author has given an annual course of lectures on elementary therapeutics at the London Hospital. His students asked him to publish them, but he never thought it worth while. But as an examiner he began to think that there might be room after all for such a book. It is curious what bad judges authors may be of the value of their own works. Here is Dr. Hutchison putting into the hands of the students not merely the cram-book which will enable them to pass their final examination, but a book which will smooth the rough pathway from pupilship to practice in a fashion unknown in modern times, and yet he reckoned it " scarcely worth while." We can only advise every student to buy the book and learn it by heart. He will not only pass his examinations, but having qualified he will find he doesn't feel half so foolish when he begins prescribing for his first patients. The scheme is to consider various diseases or symptoms and deal with the treatment under the heading of general management, diet, drugs and physical treatment. One small point of great value is the piecemeal dissection of each prescription given. We have one improvement to suggest, the illustration on p. 112 ought to be entitled " Before and after reading Hutchison's *Elements of Medical Treatment.*"

**Imhotep, the Vizier and Physician of King Zoser, and afterwards the Egyptian God of Medicine.** By JAMIESON B. HURRY, M.A., M.D. Pp. xvi., 118. Oxford University Press: Humphrey Milford. 1926.—Dr. Hurry, of Reading, so well known by his writings on vicious circles in disease, has utilised his leisure time, as well as two visits to Egypt, by collecting all that has been made out as to the story of Imhotep, whom the ancient Egyptians venerated as a great physician, and finally placed in their pantheon. How he originally won such fame as one of the first great healers is an interesting question, but it must be confessed that the facts known as to his life are few. He was the vizier and architect of Zoser 2980 B.C., the king who built the first temple of Edfu and the step pyramid of Sakkarah, the first stone pyramid. Imhotep was credited with various treatises on medicine and architecture, but practically all his writings perished about or before the Christian era. His reputation was so great that he was soon regarded as a hero or demi-god, and temples were built in his honour by the son of Cheops. Finally, under the Persians he was declared to be the god of medicine and the son of Ptah. Under the Ptolomies he was identified with Asclepios, and temples at Philæ, Thebes, Memphis, with incubation rooms for the sick, were dedicated to him. Even now endless votive statuettes of him as a demi-god are found. Many show him seated with a papyrus roll on his knees. Dr. Hurry gives us numerous beautiful photographs of these and of the temples, and an interesting account of Egyptian medicine and physiology, some of it even earlier than the time of Imhotep. The bibliography and the references to authorities for his life and cult on almost every page add greatly to the value of this charming little book.

**Diagnosis and Treatment of Venereal Diseases.** By DAVID LEES, D.S.O., F.R.C.S. Pp. xvi., 605. Edinburgh: E. & S. Livingstone. 1927. Price 15s. net.—The time is overripe for a reliable practical text-book on the Diagnosis and Treatment of Venereal Diseases from the standpoint of modern technique and with experience gained from the use of modern methods. David Lees writes from a considerable experience, and has carried out the work of advising practitioners and students with admirable skill and attention to all practical details. It is difficult to praise this book too highly, not alone for its balance in the presentment of the subject matter, but also for its admirably practical treatment, and for the omission of no detail which can make for the comprehension of the "when," "why" and "wherefore" of the tests and drugs. Where instruments are mentioned, their use is explained; where laboratory tests are invoked, these also are described and their reliability discussed. Dogmatism is nowhere overstressed, but the hand of the experienced specialist is everywhere in evidence. This book appeals to one as a safe guide, one of sound practical usefulness, and of easy reference. Scientific progress is advancing so rapidly in these subjects, that it is to be hoped that fresh editions will be kept up to date, and that this work will have a long and honoured career.

**Diseases of the Heart.** By FREDERICK W. PRICE, M.D., F.R.S. Second Edition. Pp. xvi., 534. Oxford University Press. 1927. Price 21s.—In bringing out a new edition of this well-known book, the author has introduced a good deal of new matter. For example, there is a summary of the "circus movement" view of auricular fibrillation, and the therapeutic use of quinidine sulphate is fully described. The subject of cardiac syphilis receives a more adequate notice than in the first edition; but the clinical picture of coronary thrombosis, on which so much has been written of late, is imperfectly sketched. The author evidently sees cardiac disease through the window of electrocardiography, and the book suffers from this limitation. Nevertheless, it is an honest attempt to make the new cardiology intelligible, and for this reason deserves the success which, we are told, has marked the appearance of the first edition.

**Manual of Medicine.** By A. S. WOODWARK, C.M.G., C.B.E., M.D., F.R.C.P. Third Edition. Pp. xi.,523. Oxford University Press. 1927. Price 15s.—This is one of the best-known "cram" books on medicine. The last edition appeared seven years ago, and we may well believe that—as the author says in his preface—its revision necessitated so many alterations and additions to the text that an enumeration of them is impossible. We have tested its comprehensiveness by looking up references to recent work, and while there are several notable gaps, we consider that on the whole the task of revision has been adequately fulfilled.

**Hernia and Hernioplasty.** By E. M. COWELL, D.S.O., F.R.C.S. Pp. xvi., 128. Illustrated. London: H. K. Lewis & Co. 1927. Price 9s.—This volume really consists of two portions. Under the heading of hernia, the author in the first eight chapters has collected in a concise form the results of numerous papers. Under the historical review is mentioned the fact that Mr. Steele, of Bristol, in 1873 performed the first open operation in England for a reducible inguinal hernia. Clearly-written chapters on surgical and morbid anatomy follow. The author then refers to recent work on fascial grafts. He concludes that under ordinary circumstances the conjoint tendon and Poupart's ligament will not unite when sewn together. He therefore condemns the Bassini type of operation. The second portion of the book which may be placed under the title of hernioplasty, consists of a detailed description of the author's own operation. It is profusely illustrated by photographs, but these are a little difficult to follow. In a future edition some diagrams would be helpful. The special feature of the operation consists in using two pedicled autofascial grafts cut from the external oblique aponeurosis. These flaps are stitched to Poupart's ligament beneath the cord. A special loop thread and blunt-edged needle are used. The patient gets up a week after the operation, and returns to work in one month. Operation is always performed on both sides, even if the lesion is clinically unilateral. It is not usually required in children, but may be used up to the age of eighty. The book ends with an appendix on the Workmen's Compensation Act, and on Life Assurance by Dr. Otto May.

**Epidemic Diseases of the Central Nervous System.** By ARTHUR SALISBURY MACNALTY, M.A., M.D. (Oxon). Pp. xiii., 194. London: Faber & Gwyer (The Scientific Press). 1927. Price 12s. 6d. net.—This volume contains in an expanded form the Milroy Lectures delivered before the Royal College of Physicians, London, in 1925. Three epidemic nervous diseases are considered from the epidemiological standpoint, namely cerebro-spinal fever, acute poliomyelitis, and encephalitis lethargica. These diseases are of particular interest, because we have no authentic records of previous epidemic cycles of poliomyelitis and encephalitis lethargica ; possibly we are still in the shadow of their first epidemic periods. Dr. MacNalty has written a fascinating book, one which without flattery may be compared with William Budd's famous treatise on "Typhoid Fever." But the problem offered for solution seems even more involved. In typhoid fever it was evident at an early stage of Budd's inquiry that the disease was transmitted from one patient to another. Here in the case of poliomyelitis this may still be true, for as the author says, "personal infection either direct or indirect through a carrier, is the chief if not the sole means of spread of poliomyelitis." What adds to the difficulty of the inquiry is the fact that many temporary carriers in these epidemic nervous diseases undoubtedly do not contract the disease. The meningo-coccus of Weichselbaum has been recognised as the cause of cerebro-spinal fever, and it is known that in epidemic periods carriers of the mening-ococcus are widespread among the population of the area affected. Yet even in cerebro-spinal fever we do not know the nature of "that mysterious catalytic agent of infection which is termed susceptibility." Our ignorance of the infective factors necessary to produce the diseases acute poliomyelitis and encephalitis lethargica is even greater. Dr. MacNalty does not solve the riddle once and for all, as Budd succeeded in doing for typhoid, but he presents the problem and the facts bearing on it in a most interesting and readable way.

**The Health of the Child of School Age.** Edited by Sir THOMAS OLIVER, M.D., LL.D., D.C.L., F.R.C.P. Pp. viii., 204. Oxford University Press. 1927. Price 6s.—This book is designed, as stated in the Foreword, particularly for teachers. The chapter on Diet in Schools is excellent, and the school is fortunate which possesses a doctor who combines such sound principles and sound practice. In the chapter on Infectious Diseases, we find the following statement: "Should the number of cases of scarlatina threaten to become large, the medical officer would have reason to suspect the existence of some insanitary condition in or near the school premises." For many years epidemiologists have been trying to remove from the public mind the idea that our ordinary zymotic diseases may be caused by insanitary conditions, and we are sorry to see this doctrine propagated. On page 109 parents are recommended to expose their healthy children at an early age to the infection of measles. We protest emphatically against this teaching. Measles is a very grave disease, responsible for a heavy mortality, greater by far than that of diphtheria and scarlatina together, and every year by which the attack is postponed diminishes its risks. The serious sequelæ which may ensue from measles are well set out on page 125, in the excellent chapter on Affections of the Nose, Throat and Ear in this book. We are rather surprised to see no allusion to the Schick method of immunisation against diphtheria. In the chapter entitled "The Eye Troubles of School Life," errors of refraction are not dealt with. This is a most serious omission, and it cannot be regarded as a consolation to the purchaser of the book that the author has written or lectured on the subject elsewhere. On the other hand, trachoma, glare-asthenopia, and hay-fever are dealt with at some length. These are not very common eye diseases of English school children. In the chapter on Preventable Deformities the author states that dried milk has only half the anti-scorbutic value of fresh milk. This is correct in regard to milk dried by the older spray process, but is not true in regard to the modern roller (Just-Hatmaker) method of drying. The statement is to be regretted, as dried milk has a definite and well-recognised value, especially in times of epidemic diarrhœa. In the section entitled "Prevention of Tuberculosis Deformities" no mention is made of bovine infection. We are rather surprised that the question of mentally subnormal children is omitted from this book, and also that there is no article on the important subject of heart disease and rheumatic infections of childhood. On the whole we feel that the subject matter has not been very wisely selected, and that there are some statements which require alteration.

**Exophthalmic Goitre.** By John Eason, M.D., F.R.C.P.E. Pp. 215. 19 plates and other illustrations. Edinburgh : Oliver & Boyd. 1927. Price 12s. 6d.—This should be a valuable work. It contains the author's views on the latest researches and methods of treatment in this disease, together with an extensive list of references. The work of Williamson and Pearse on the anatomical structure of the thyroid gland, and their conception of its secretory phases, are considerably referred to, though the interpretation of the context is here often difficult to follow. The author discourses at some length on the autonomic nervous system, and outlines its connections with the endocrine glands and the thyroid in particular. There is an excellent description of the clinical manifestations of the disease, including an illuminating discussion of the eye signs ; the writer disputes the presence of dilation of the pupils, a condition hitherto generally accepted. Etiological features and pathogenesis are fully discussed, an inborn susceptibility being apparently a *sine qua non*, a great majority of cases being precipitated by some mental crisis, which may often be concealed. Those afflicted with this malady will never do great things : "no outstanding man or woman appears to have suffered from the disease during the eighty years since it became generally recognised." The conception of a certain physical type of individual, peculiar to this disease, being a subject of congenital endocrinopathy and therefore prone to autonomic imbalance is certainly intriguing, and the susceptibility of certain people to small doses of thyroid extract is pointed out in this connection. The significance of blood pressure readings is fully discussed, and interesting figures are included to indicate the relationship of pulse pressures to basal metabolic rates in Graves's disease, the explanation of the increased pulse pressures being ingenious and acceptable. The difficult question of automatic imbalance with sympathetic preponderance and its differentiation from pure Graves's disease is alluded to, but without much enlightenment to the reader. From the chapter on prognosis one gathers much help in an effort to foresee the ultimate trend of a case. In a very adequate chapter on treatment concluding the work medical treatment under "ideal conditions" is affirmed to be the sheet anchor ; there is an interesting comparison of this disease and diabetes, and insulin as a factor in treatment is reviewed and its use here carefully outlined ; the fate of the blood sugar and the emaciation occurring in the course of the disease are discussed at length. The indications for administering iodine are outlined, its dangers pointed out, and the author maintains that it is invaluable in the preparation of a case, and that " post-operative death can be prevented " by its previous judicious exhibition. Thyroidectomy is regarded as a dangerous procedure, and only to be ventured upon in certain well-defined circumstances. Although the style of the writer is somewhat heavy, some very interesting diagrammatic tables are inserted, and the general finish and print of the book is excellent.

**The Heart and its Diseases.** By C. W. CHAPMAN, M.D., M.R.C.P. Pp. xii., 216. Illustrated. Edinburgh: E. & S. Livingstone. 1927. Price 8s. 6d.—This is a book which contains a great deal of subject-matter in small compass. It is clearly set out, well printed and is a very readable book. It is perhaps too condensed to be of use to students, but should be exceptionally valuable to general practitioners who are too busy to read long text-books on the subject. The chapter on Rheumatism and Heart Disease and that of Heart Disease in Children contain a very good summary of the recent teaching, and that on treatment, though very short, outlines very clearly the methods of treatment, though more emphasis might have been laid on the necessity of continuing the administration of digitalis in auricular fibrillation after the rate has been slowed by this drug, a point which is still not understood by many practitioners. It is certainly a book which will fulfil a useful purpose.

**Chronic Intestinal Stasis.** By A. C. JORDAN, C.B.E., M.D., M.R.C.P. 2nd Edition. Pp. xv., 230. Oxford University Press. 1926. Price 21s.—Dr. A. C. Jordan's second edition of his book on chronic intestinal stasis is a valued addition to our knowledge of this branch of Radiology. He has had a unique experience of intestinal radiology, and has done an immense amount of work along these lines, and one is very glad to have his opinions and experience in print. The present book brings the subject well up to date, and should be in the library of all interested in this subject.

**Cystoscopy.** By J. B. MACALPINE, F.R.C.S. Pp. xvi., 284, 181 illustrations, 12 coloured plates. Bristol: John Wright & Sons Ltd. 1927. Price 25s. net.—This should prove a valuable book to those who propose to take up the study of cystoscopy seriously. It is obviously compiled by one whose experience is extensive and whose observation is accurate; but while not being exhaustive in its treatment of the subject, it is eminently suitable for those about to embark on genito-urinary work.

**Treatment of Venereal Disease in General Practice.** By E. T. Burke, D.S.O., M.B., Ch.B. Pp. xiv., 162., 8 illustrations. London: Faber & Gwyer. 1927. Price 5s.—This little book, containing only 158 pages, certainly performs the object of its creation, *i.e.* a brief, clear description of a scheme for the routine treatment of venereal diseases such as can be carried out by the general practitioner. It is delightfully written, and should be most useful to all practitioners; even those who do not wish to treat venereal diseases would find it helpful in advising their patients as to the amount and length of treatment they should undergo to be cured. The book is divided into two parts—the treatment of syphilis, and the treatment of gonorrhœa, the final chapter of each being "obiter dicta" which should be committed to memory by all practitioners, and students.

**A Handbook of Diseases of the Stomach.** By S. Ward, M.D., M.R.C.P. Pp. x., 387., 32 illustrations. Oxford University Press. 1927. Price 16s. net.—This work is worthy of close study: Dr. Ward bases his views on practical experience, and has interesting views on the physiology of gastric sensation. He goes far to dispel the illusions which may exist with regard to the infallibility of any one method of investigation of gastric disorder; it is refreshing to come upon an author who bases his observations on sound common sense. The conclusions, false and otherwise, that may be drawn from the various means at our disposal for the diagnosis of gastric disease are thoroughly criticised, and their proper value assessed; the several fashions of treatment are dealt with in the same way, perhaps somewhat severely, but, at any rate, these procedures are narrowed down to those few which the author has found by experience to be of practical value and not based on the extravagant hypotheses of theoretical enthusiasts. As an example, whilst admitting that an abdominal belt may comfort the gastroptotic, its actual influence in raising the position of the stomach is shown by comparative radiographic pictures to be practically nil. In the matter of diet the effects of the various methods of cooking are particularly well dwelt upon, and it is a relief to hear that one is not lost in respect of alimentary salvation, or becoming bankrupt in the vitamines, by not eating wholemeal bread. Bismuth is assailed time and again, for whilst its help in the relief of symptoms is acknowledged, it is accused of doing more ultimate harm than good, except in a few well-defined conditions. Stomach rest should be the main principle of treatment for the majority of the various dyspepsias. This, with gastric lavage and duodenal feeding, is urged to promote the healing of gastric ulcer; surgical inteference in this condition is strongly deprecated. In regard to malignant disease of the stomach, however, "whenever there is any reasonable suspicion that cancer is present, and it cannot be definitely excluded, exploratory laparotomy should be advised at the earliest possible moment." The author regards X-ray treatment as a dangerous procedure and a useless remedy for this condition; radium also he considers an impracticable method to employ. On the other hand, he claims markedly beneficial results from large intravenous doses of colloidal selenium. This book is wholesome reading, and the author is to be congratulated on his courageous handling of the present day situation in regard to gastric disease.

**Venereal Disease: Its Prevention, Symptoms and Treatment.** 3rd Edition. By H. W. BAYLY, M.C. Pp. xvi., 242., 74 illustrations and 3 plates. London: Faber & Gwyer. 1927. Price 10s. 6d. net.—A small handbook suitable for the general practitioner or student. It is very well arranged, the type excellent and illustrations good. It brings to the reader a sharp and clear view of modern treatment, and indicates in a most thorough manner those points which are so apt to be overlooked in the routine investigation adopted by so many busy practitioners. The chapter on prophylaxis is excellent. The inexpensive nature of the book should commend it to a large public.

**The Life and Work of Sir Patrick Manson.** By P. H. MANSON-BAHR, D.S.O., M.D., F.R.C.P., and A. ALCOCK, C.I.E., LL.D. Aberd., F.R.S., I.M.S. (retired). Pp. x., 273. 12 plates. London: Cassell & Co. Ltd. 1927. Price 16s.—This book is an extremely interesting and inspiring biography, giving a full insight into the life and work of that great man, "The Father of Tropical Medicine," as he has been so appropriately named. For twenty-three years he lived in China, on the island of Formosa, on the mainland, and at Arnoy and in the British Settlement of Hong Kong respectively. It would be difficult to find a less favourable environment for research work, cut off as he was from contact with the scientific world and busy with the routine work of a large native hospital, Port health officer and an extensive private practice; but he found time, despite these difficulties, to carry out his epoch-making discoveries in filarial disease. During the latter part of his life spent in London, Manson's mosquito-malaria-theory was definitely established by Sir Ronald Ross in India. The details of this are strikingly brought out with the help of numerous extracts from letters, which show the fine, generous and unselfish character of Manson in offering Ross the results of his knowledge and experience, the utmost encouragement that he gave him, and the firm faith he had in the ultimate result. Manson's arresting personality, his patience, faculty of judgment, the result of keen observations, and his skill in divining the life history of parasites are all well brought out by the authors. The book is not only interesting and inspiring as a biography, but is instructive, especially in the history of malaria and filariasis, which is more fully dealt with than in most text-books on tropical diseases.

**Therapeutic Malaria.** By G. de M. Rudolf, M.R.C.S., L.R.C.P., D.P.H., D.P.M. Pp. xi., 223, 55 illustrations, 2 coloured plates. London: Humphrey Milford. 1927. Price 12s. 6d. net.—The theory of treating one disease by the induction of an intercurrent disease is a fascinating subject, and as the author points out, dates from ancient times. The improvement and even the dramatic recovery of patients suffering from the acute psychoses following high fever from accidental intercurrent infection by the pneumococcus, streptococcus, etc., is a frequent experience. Whether this change is due to leucocytosis, high temperature, metabolic changes, or the formation of antibodies, or any of these combined, remains unproven; and Dr. Rudolf discusses each theory in turn. The theory of leucocytosis has been made practical use of in this direction by the injection of substances both organic and inorganic to produce this effect. Though experiments of this nature have been tried on all forms of mental disorder, general paralysis has been for several years the most favoured. While the occurrence of remissions in general paralysis without any treatment or with other treatment than induced malaria is frequent, the author gives details of the very strong evidence both from his own experience and that of other workers that remissions occur far more frequently following malaria, and, moreover, can confidently be expected in a high per centage of cases. There are few mental hospitals where this treatment is not now being carried out. The results amply justify it, particularly in a disease which up to the present time has been considered fatal. Dr. Rudolf divides his book into two parts—

historical and practical. The practical part is well arranged, and deals clearly and thoroughly with the technique, the parasite and inoculation, the blood changes and the temperature chart, and points out the dangers to be guarded against. The volume forms an interesting study both for the student and as a guide to those carrying out the treatment. The temperature charts are clear and easy to follow, and there are two admirably coloured plates dealing with the blood. The numerous references at the end are listed in alphabetical order, and are not crowded into small print as is so often the case.

**Urinary Surgery.** By W. K. IRWIN, M.D., F.R.C.S.E. 2nd edition. Pp. viii., 271. London: Ballière, Tindall & Cox. 1927. Price 10s. 6d.—This book, dealing with urological surgery from the clinical standpoint of the general practitioner, appears to be concise and accurate. While by no means exhaustive in its treatment of the subject, it should act as a useful guide to the practitioner in judging his ability to deal with any condition or the advisability of handing on the patient to those more versed in the particular subject. The methods of examination of the patient are dealt with adequately, and the treatment also in the less complicated cases. The chief symptoms have been described in relation to their cause and treatment, and this from the general practitioner's point of view has a definite advantage over a regional arrangement of the subject. It should be found of value to those embarking on general practice.

